# An alternative strategy for balancing profit maximization and risk reduction

**DOI:** 10.1371/journal.pone.0348577

**Published:** 2026-05-15

**Authors:** Youssef El Khatib, Farangiz Mukhamedova

**Affiliations:** 1 Department of Mathematical Sciences, United Arab Emirates University, Al-Ain, United Arab Emirates; 2 Heriot-Watt University Dubai, Dubai, United Arab Emirates; Incheon National University, KOREA, REPUBLIC OF

## Abstract

Portfolio diversification is a central theme in modern investment theory. We revisit the classic return–risk trade-off and propose an alternative objective $Q_{\lambda }(w)=\mu^{⊤}w+\lambda (w^{⊤}\Sigma w{)}^{-1/2}$ that balances higher expected returns with a direct penalty on portfolio volatility via the inverse standard deviation. This objective belongs to the axiomatic class of mean–variance preferences (as formalised by [1] for additively separable forms) and admits tractable solutions, including a closed-form characterisation in the two-asset case. In rolling out-of-sample backtests on standard Fama–French equity portfolios with realistic trading costs and long-only constraints, $Q_{\lambda }$ delivers statistically significantly lower realised volatility (paired t-test *p* < 0.01) and shallower maximum drawdowns than the Sharpe-maximising benchmark, while maintaining average performance comparable to Markowitz, equal-weight, and risk-parity strategies. These advantages persist across major stress episodes (GFC, COVID-19, 2022 rate shock) and are robust over a wide range of the trade-off parameter λ. The results position $Q_{\lambda }$ as a pragmatic alternative for investors who value smoother wealth paths and robust downside protection.

## Introduction

Portfolio diversification remains one of the most effective strategies for managing investment risk. The modern portfolio theory provides a solid mathematical framework for understanding diversification and optimizing portfolios. Still, practical considerations—such as the choice of asset classes and the correlation between them—are crucial for successful implementation. As investment opportunities become increasingly complex, a well-diversified portfolio can provide a more stable and predictable return, making it a foundation of effective wealth strategy [1].

Modern Portfolio Theory (MPT), introduced by [2], remains a cornerstone of diversification strategies. However, its limitations have led to the development of alternative approaches. The literature contains various studies and techniques that collectively contribute to a more nuanced understanding of portfolio optimization, considering factors such as risk-adjusted returns, financial literacy, asset correlations, alternative investments, and the impact of investment horizons on diversification benefits.

The literature reveals several major research directions in portfolio diversification studies, see for example [3] for a comprehensive bibliometric review of 242 articles published between 1974 and 2022. The evolution of portfolio diversification amid increasing market integration and the rise of new asset classes such as cryptocurrencies is traced [4]. Principal research directions include: (i) portfolio diversification fundamentals, (ii) diversification strategies, (iii) geographical focus on emerging markets [5], and (iv) diversification benefits with specific assets [6].

A principal research direction on portfolio diversification encompasses both technical foundations—such as currency exposure and asset allocation—and non-financial determinants including education, home bias, and cultural influences, with recent works also addressing the effects of major events like COVID-19 [7–9]. Another direction focuses on strategies, and it centers on optimization techniques and comparative model performance, evaluating frameworks such as mean–variance and Black–Litterman, as well as considerations of skewness and strategies like variance minimization [10–12]. Additionally, many research papers on portfolio diversification study the benefits with specific asset classes, and they examine diversification advantages derived from assets including equities, commodities, and cryptocurrencies, emphasizing decorrelation effects during crises and improvements in risk-adjusted returns [4,13,14]. Geographical PD focuses on emerging markets, examining diversification opportunities in emerging, frontier, and regionally integrated markets, noting reduced benefits due to globalization, crises, and time-varying correlations [15–17].

The literature on portfolio diversification is vast and multi-faceted, addressing a range of theoretical and practical questions fundamental to investment science. As comprehensively surveyed in Koumou’s review, research on portfolio diversification can be broadly grouped into several key directions, each representing a distinct thematic concern found throughout the academic discourse [18].

Recent studies have also examined dynamic international spillovers and quantile-based perspectives in the formation of investor preferences [19], and [20]. Our contribution differs by proposing a simple, interpretable, additively separable objective that directly penalises total volatility and demonstrates superior out-of-sample risk control on standard test assets. Our additive inverse-risk penalty differentiates from ratio-based objectives by directly penalizing volatility, offering better tail-risk control amid spillovers. We build on quantile perspectives for economic relationships, enhancing understanding of tail-dependent preferences.

A first significant research avenue explores diversification through the lens of the law of large numbers, focusing on how holding a greater number of assets reduces unsystematic risk. Several papers demonstrate that the benefits from merely increasing asset quantity may reach diminishing returns, raising questions about the optimal number of assets required for adequate risk reduction [21–24]. Within this theme, empirical and theoretical contributions examine both the traditional strength and the limits of the law of large numbers, especially when asset returns are not independent or identically distributed.

A second major direction in the literature centers on the role of correlation among asset returns. Diversification is most effective not simply when portfolios contain many assets, but when these assets exhibit low or negative correlations, an idea traced to the roots of modern portfolio theory. Recent contributions analyze how changing correlation structures—across asset classes, geographies, or time—alter the attainable frontier of risk-return trade-offs for diversified portfolios [25–28]. The literature increasingly focuses on understanding and managing correlation risk, as periods of market turmoil have revealed unexpected increases in correlations that reduce diversification benefits.

A third identified research stream deals with the intersection of diversification and asset pricing models, primarily the Capital Asset Pricing Model (CAPM). Studies in this area investigate how diversification aligns with or challenges the foundational assumptions of CAPM and related frameworks, especially the notion that only systematic risk should earn a risk premium [29–32]. Researchers here bridge measurement of diversification with asset pricing implications for portfolio construction, performance attribution, and evaluation.

Finally, the literature has advanced into more sophisticated approaches such as risk contribution and risk parity, exploring how assets contribute to total portfolio risk, rather than simply targeting equal capital allocation. This burgeoning field studies how to construct portfolios where risks are more evenly distributed across assets, leading to potentially greater resilience and different performance signatures than traditional weighting schemes [33,34,35,36]. These works frequently assess risk-based allocation as an alternative to mean-variance optimization, with both theoretical justification and empirical validation.

Recent research in portfolio diversification has explored various approaches to optimize investment strategies across different asset classes and market conditions. For instance, [37] investigated the diversification benefits of cryptocurrencies before and during the COVID-19 pandemic, finding that their inclusion can improve portfolio performance, particularly during crisis periods. The authors of [38] conducted experimental studies on diversification behavior, examining how individuals make portfolio choices when presented with correlation information. The work in [39] focused on portfolio diversification and value at risk analysis under heavy-tailed distributions, challenging traditional assumptions about risk. On the other hand, [40] explored global portfolio diversification for long-horizon investors, considering the impact of permanent cash flow shocks and transitory discount rate shocks on asset prices and returns. The study in [41] compared the out-of-sample performance of various portfolio strategies, including the naive 1/N diversification rule, finding that many optimized models struggle to consistently outperform this simple approach. The diversification potential of alternative investments is examined in [42] such as commodities and hedge funds, in institutional portfolios. In [43], it is provided a comprehensive review of modern quantitative portfolio construction and optimization techniques, discussing both theoretical foundations and practical implementations. Another works such as in [44] introduced a novel approach using two-sample graph kernel methods to assess portfolio diversification, allowing for robust testing and comparison of various screening criteria or optimal investment strategies. This method is particularly useful for practitioners developing ESG factor investing-based models, ESG ETF funds, and impact funds.

The literature discusses mean–variance optimization as a means of maximizing diversification benefits [45], as well as comparative strategies such as variance minimization versus return maximization [46]. These approaches implicitly connect to the concept of expected utility maximization, rooted in Markowitz’s seminal portfolio selection theory [2]. The paper by [1] presents necessary and sufficient preference-based axioms for the existence of mean-variance (MV) utility, where random variables with the same mean and variance are equally desirable, without explicitly specifying conditions on variances beyond mean values. It develops three axiom systems tailored to simple random variables, those with bounded supports, and variables bounded in preference, ensuring MV utility representation in each case. Furthermore, it investigates axioms for four additively separable forms of mean and variance in the utility function, incorporating variants of constant risk aversion to derive linear, square root, and weighted sum models. This work advances the understanding of MV analysis as an alternative to expected utility maximization in decision making under risk. Advanced diversification techniques include scenario testing, which simulates portfolio performance under different economic conditions, and asset correlation analysis, which examines how different investments move in relation to each other [47], and in [46] and [48] a method to maximize risk-adjusted returns is used for variance minimization.

Taken together, these diverse research trajectories reflect the complexity and dynamism of portfolio diversification theory, as highlighted by Koumou’s comprehensive review. The themes of the law of large numbers, correlation structures, asset pricing models, and risk contribution approaches each occupy a prominent place in the academic and practitioner literature.

Against this backdrop, we revisit the return–risk trade-off and ask whether a simple, interpretable objective can balance profit maximization with robust downside control more reliably than ratio-based rules. We propose an additively separable objective, $Q_{\lambda }(w)=\mu^{⊤}w+\lambda \;(w^{⊤}\Sigma w{)}^{-1/2},$ that rewards expected return while penalizing total risk through the inverse standard deviation. This form lies within the axiomatic envelope of mean–variance preferences [1], and we provide a closed-form characterization in the two-asset case together with a practical solution for the general constrained problem.

Our contributions are threefold. *First*, we establish the theoretical underpinnings of $Q_{\lambda }$ and derive tractable optimality conditions, clarifying how the inverse-risk term tempers fragile, concentrated allocations typical of Sharpe maximization. *Second*, we design a transparent rolling backtest (long-only, monthly, realistic trading costs) on standard Fama–French test assets, enabling full reproducibility. *Third*, we document that $Q_{\lambda }$ delivers lower realized volatility and shallower drawdowns than a Sharpe-maximizing portfolio, while achieving average performance comparable to Markowitz, equal-weight, and risk-parity baselines; these patterns persist across major stress episodes and are stable over a range of λ values.

This study addresses: What is an effective alternative to ratio-based optimization for balancing returns and risk? This question is important for investors seeking stability in volatile markets. We approach it via theoretical derivations and empirical backtests with frictions, persuasive due to out-of-sample robustness and statistical significance.

The rest of the paper proceeds as follows. Section details the data and out-of-sample protocol and reports the empirical results. Earlier sections introduce the optimization framework, provide the two-asset solution and general solver, and relate our objective to mean–variance utility. Section Conclusion concludes with practical implications, limitations, and directions for extending $Q_{\lambda }$ (e.g., light position caps, duration guardrails, or semideviation variants).

## Portfolio optimization problem

The concept of portfolio optimization lies in the expected return of a portfolio, which is the weighted sum of the expected returns of its constituent assets, and the risk (or volatility) of the portfolio which is measured by the variance (or standard deviation) of the portfolio’s returns. The goal is to find a portfolio that maximizes expected return for a given level of risk or minimizes risk for a given level of expected return. Considering *n* assets, then the portfolio’s expected return, *P*_*R*_, is given by:


$$\begin{array}{c} P_{R}=w_{1}{A_{1}}_{R}+w_{2}{A_{2}}_{R}+\cdots +w_{n}{A_{n}}_{R} \end{array}$$

where *w*_*i*_ is the weight of asset *i* in the portfolio, and ${A_{i}}_{R}$ denotes the expected return of asset *A*_*i*_.

The portfolio’s risk (variance), $\sigma_{P}^{2}$, is given by:


$$\begin{array}{c} \sigma_{P}^{2}=\sum_{i=1}^{n}w_{i}^{2}\sigma_{i}^{2}+\sum_{i\neq j}w_{i}w_{j}\text{Cov}({A_{i}}_{R},{A_{j}}_{R}) \end{array}$$

where $\sigma_{i}^{2}$ is the variance of asset *A*_*i*_ and $\text{Cov}({A_{i}}_{R},{A_{j}}_{R})$ is the covariance between the returns of assets *A*_*i*_ and *A*_*j*_.

The Markowitz portfolio optimization problem can be formulated as follows:

**Minimize:**


$$\begin{array}{c} \sigma_{P}^{2}=\sum_{i=1}^{n}w_{i}^{2}\sigma_{i}^{2}+\sum_{i\neq j}w_{i}w_{j}\text{Cov}({A_{i}}_{R},{A_{j}}_{R}) \end{array}$$

**Subject to:**

- The target R^*^ return constraint:
  $$\begin{array}{c} P_{R}=\sum_{i=1}^{n}w_{i}{A_{i}}_{R}\geq R^{*} \end{array}$$ (1)
- The budget constraint:
  $$\begin{array}{c} \sum_{i=1}^{n}w_{i}=1 \end{array}$$ (2)
- Non-negativity constraints:
  $$\begin{array}{c} w_{i}\geq 0,\;\forall i=1,\ldots ,n \end{array}$$ (3)

Where R^*^ is the expected return that an investor aims to achieve with their portfolio. This optimization problem minimizes the portfolio risk (variance) while ensuring that the expected return meets or exceeds a specified target R^*^. The constraints ensure that the portfolio weights sum to 1 and are non-negative, representing a long-only portfolio without leverage.

Modern portfolio theory formalizes the trade‑off between expected return and variance [2]. Empirically, however, diversification benefits are state‑dependent: correlations tend to rise in crises [49], and some episodes of apparent “contagion” are better explained by shifts in interdependence once heteroskedasticity is accounted for [50]. These facts caution against rules that rely too heavily on point estimates of second moments.

Out of sample, simple benchmarks such as 1/*N* are often difficult to dominate across a wide range of datasets, windows and priors [51]. In practice, many workflows temper estimation error through shrinkage, views, and regularization; Black–Litterman integrates investor views with equilibrium priors [52], while equal risk contribution distributes total risk across positions [53]. A comprehensive survey of practical challenges and trends is provided by [43]. For long‑horizon investors, predictable components in returns and volatility further tilt optimal allocations [54]. A recent bibliometric map of the literature documents the evolution of these strands since the 1970s [3].

Many portfolio rules effectively optimize either (i) a *quadratic* mean–variance utility or (ii) a *ratio*, e.g., Sharpe‑style objectives or the risk‑adjusted criterion of [46]. Ratio objectives can become fragile when estimated volatilities are transiently understated, inducing concentrated bets; strongly risk‑averse quadratic utilities, by contrast, may forgo return premia.

The optimization problem proposed by [46] focuses on maximizing the risk-adjusted return of a portfolio. The formulation can be described as follows:

**Objective:** Maximize the risk-adjusted return of the portfolio, defined as:


$$\begin{array}{c} R_{adjusted}=\frac{P_{R}}{\sigma_{P}} \end{array}$$ (4)

**Subject to:** constraints (2–3)

The optimization approach (4) contrasts with traditional methods that typically aim to minimize variance while achieving a target return. It emphasizes a holistic view by integrating risk and returns in a single optimization framework, making it more suitable for rational investors looking to maximize their returns relative to the risks taken. This formulation allows for flexibility in portfolio construction, accommodating various investment strategies while focusing on enhancing overall performance through effective asset allocation.

### Alternative objective function

We build on the existing foundation by introducing a novel objective function that explicitly balances return maximization and variance minimization, extending the axiomatic underpinnings of mean-variance utility formalized by [1]. Unlike traditional mean-variance optimization, which often prioritizes variance reduction at the expense of forgone returns [45], or simplistic 1/N rules that overlook asset-specific risks [41], our framework incorporates adaptive weighting to enhance robustness across market regimes, including those with alternative assets and long-horizon shocks [40]. This approach is motivated by persistent gaps in the literature: while strategies like downside risk constraints [11] and correlation-aware allocations [42] mitigate some limitations, they rarely reconcile in-sample efficiency with out-of-sample stability in volatile, non-normal environments.

More precisely, we introduce a new objective as the sum of the expected return of the portfolio *P*_*R*_ and the inverse of the standard deviation of the portfolio $\sigma_{P}$, that is our objective function is defined as follows:


$$\begin{array}{c} Q_{\lambda }=P_{R}+\frac{\lambda }{\sigma_{p}}. \end{array}$$ (5)

The objective function can be explicitly written as $Q_{\lambda }(w)=\mu^{⊤}w+\lambda \;(w^{⊤}\Sigma w{)}^{-1/2},$ is *additively separable* in mean and a decreasing function of variance and lies within the axiomatic envelope for mean–variance preferences [1]. Intuitively, the inverse-risk term imposes a steeper marginal penalty at low volatility, discouraging fragile concentration without fully flattening exposures as risk-parity rules may do.

#### Mean–variance classification.

While the proposed objective is not derived from quadratic utility, it belongs to the broader axiomatic class of additively separable mean–variance preferences characterized by Nakamura (2015). In this representation, preferences depend solely on the first two moments and admit additive separability between mean and a monotonic transformation of variance. The inverse standard deviation term preserves mean–variance ordering while introducing nonlinear curvature that differs from traditional quadratic utility. This imposes a steeper marginal penalty at low volatility levels, meaning that incremental reductions in volatility are rewarded more aggressively when the portfolio is already stable, encouraging diversification even in low-risk regimes. Economically, this reflects investor preferences for robustness against tail events, as supported by generalized mean-variance frameworks [1,2] and tail-dependent perspectives [19,20].

## Optimal Two-Asset Allocation via the Sum of Return and Inverse Risk Maximization

In this section, we solve the optimization problem defined by the objective function (5) subject to constraints (2–3) in the case of a portfolio with two assets, where we have assumed λ=1. From now on, we assume that the portfolio contains only two assets, *A*_1_ with an expected return of μ, and *A*_2_ with an expected return of ν. The weight allocated to asset *A*_1_ is denoted by *x*, and the weight for *A*_2_ is *y*.If the correlation is ρ and the standard deviations of the returns of *A*_1_ and *A*_2_ are α and β, respectively, then the expected return of the portfolio can be written as


$$\begin{array}{c} f(x,y)=\mu x+\nu y. \end{array}$$

The variance of the portfolio (risk) is given by


$$\begin{array}{c} \Omega (x,y)=\alpha^{2}x^{2}+\beta^{2}y^{2}+2\rho \alpha \beta xy, \end{array}$$

where α,β>0, |ρ|≤1. The present paper proposes an alternative approach to studying risk-return optimization by introducing the sum of return and inverse risk as an objective function. Our objective function is given by: $Q(x,y)=f(x,y)+\frac{1}{\sqrt{\Omega (x,y)}}.$ The optimization problem is to determine the optimal weights (*x*,*y*) such that *Q*(*x*,*y*) is maximized, subject to constraints (2–3): *x* + *y* = 1 and x,y≥0. We first write the main result in the following theorem that provides the solution of our optimization problem.

**Theorem 1.** *Let*


$$\begin{array}{c} \xi =\alpha^{2}+\beta^{2}-2\rho \alpha \beta , \end{array}$$ (6)

$$\begin{array}{c} \eta =\rho \alpha \beta -\beta^{2}. \end{array}$$ (7)

*Assume that* μ>ν, β≥α*, and*


$$\begin{array}{c} 0\leq \beta^{2}-\frac{\sqrt{\xi }}{\sqrt{3}(\mu -\nu )}<\frac{\eta^{2}}{\xi }. \end{array}$$ (8)

*Moreover, consider the function*


$$\begin{array}{c} g(t)=(t-A{)}^{3}-Bt,\;\text{with }A=\alpha^{2}\beta^{2}(\rho -1)\leq 0,\;B=\frac{\xi^{3}}{(\mu -\nu {)}^{2}}\geq 0, \end{array}$$ (9)

*and let* $\tau_{2}$ *be the root of g(t) satisfying*


$$\begin{array}{c} t_{1}:=A-\sqrt{\frac{B}{3}}\leq \tau_{2}\leq t_{2}:=A+\sqrt{\frac{B}{3}}. \end{array}$$

*Then, the solution of the optimization problem*


$$\begin{array}{cc} \text{Maximize:}\; & Q(x,y)=f(x,y)+\frac{1}{\sqrt{\Omega (x,y)}}\\ \text{subject to:} & \;x+y=1\\ & x,y\geq 0 \end{array}$$ (10)

*is*


$$\begin{array}{c} x^{*}=\left\{ \begin{array}{c} \frac{-\eta }{\xi }\;\text{if }\mu =\nu \\ \frac{\sqrt{\tau_{2}}-\eta }{\xi }\;\text{if }\mu \neq \nu \end{array}\right. \end{array}$$ (11)

$$\begin{array}{c} y^{*}=1-x \end{array}$$ (12)

The proof of the theorem can be obtained as a combination of the two next propositions.

**Proposition 1** *The optimization problem (10) is equivalent to*


$$\begin{array}{cc} & \text{Maximize:}\;G(x)=(\mu -\nu )x+\nu +\frac{1}{\sqrt{\xi x^{2}+2\eta x+\beta^{2}}}\\ & \text{subject to:}\;0\leq x\leq 1 \end{array}$$ (13)

*where*


$$\begin{array}{c} \xi =\alpha^{2}+\beta^{2}-2\rho \alpha \beta , \end{array}$$ (14)

$$\begin{array}{c} \eta =\rho \alpha \beta -\beta^{2}. \end{array}$$ (15)

**Proof.** In what follows, without loss of generality, we may assume that μ≥ν and β≥α. Now, replacing *y* = 1 − *x*, then the function *Q* is reduced to


$$\begin{array}{c} G(x):=Q(x,1-x)=(\mu -\nu )x+\nu +\frac{1}{\sqrt{\Omega (x,1-x)}}. \end{array}$$

One can calculate that


$$\begin{array}{cc} \Omega (x,1-x) & =\alpha^{2}x^{2}+\beta (1-x{)}^{2}-2\rho \alpha \beta x(1-x)\\ & =(\underset{\xi }{\underbrace{\alpha^{2}+\beta^{2}-2\rho \alpha \beta }})x^{2}+2(\underset{\eta }{\underbrace{\rho \alpha \beta -\beta^{2}}})x+\beta^{2}\\ & =\xi x^{2}+2\eta x+\beta^{2}. \end{array}$$

Hence,


$$\begin{array}{c} G(x)=(\mu -\nu )x+\nu +\frac{1}{\sqrt{\xi x^{2}+2\eta x+\beta^{2}}}. \end{array}$$

This concludes the proof of the proposition.

The next proposition solves the optimization problem 2 given by (13).

**Proposition 2** *Assume that* μ>ν, β≥α, *and*


$$\begin{array}{c} -\frac{3}{2}A<\sqrt{\frac{B}{3}}\leq \xi \beta^{2}, \end{array}$$ (16)

*where*


$$\begin{array}{c} A=\alpha^{2}\beta^{2}(\rho^{2}-1)\leq 0,\;B=\frac{\xi^{3}}{(\mu -\nu {)}^{2}}\geq 0. \end{array}$$

*Moreover, consider the function*


$$\begin{array}{c} g(t)=(t-A{)}^{3}-Bt, \end{array}$$ (17)

*and let* $\tau_{2}$ *be the root of g(t) satisfying*


$$\begin{array}{c} t_{1}:=A-\sqrt{\frac{B}{3}}\leq \tau_{2}\leq t_{2}:=A+\sqrt{\frac{B}{3}}. \end{array}$$

*Then, the solution of the optimization (13) is*


$$\begin{array}{c} x^{*}=\left\{ \begin{array}{c} \frac{-\eta }{\xi }\;\text{if }\mu =\nu \\ \frac{\sqrt{\tau_{2}}-\eta }{\xi }\;\text{if }\mu \neq \nu \end{array}\right. \end{array}$$ (18)

**Proof.** Due to our assumption (β≥α), we notice that


$$\begin{array}{c} \xi >0,\;\eta =\beta (\rho \alpha -\beta )\leq 0. \end{array}$$

Let us find the critical points of the function *G*(*x*) of the optimization problem (13):


$$\begin{array}{c} G^{\prime }(x)=0, \end{array}$$

which yields


$$\begin{array}{c} (\mu -\nu )-\frac{\xi x+\eta }{\sqrt{(\xi x^{2}+2\eta x+\beta^{2}{)}^{3}}}=0. \end{array}$$

Hence,


$$\begin{array}{c} (\mu -\nu )\sqrt{(\xi x^{2}+2\eta x+\beta^{2}{)}^{3}}=\xi x+\eta . \end{array}$$ (19)

We point out that if μ=ν, then the critical point is


$$\begin{array}{c} x_{0}=-\frac{\eta }{\xi }. \end{array}$$

Therefore, in the sequel, we always assume that μ>ν. Hence, the equation (19) is reduced to


$$\begin{array}{c} (\mu -\nu {)}^{2}(\xi x^{2}+2\eta x+\beta^{2}{)}^{3}=(\xi x+\eta {)}^{2}. \end{array}$$ (20)

Now, changing to the variable $\tau =(\xi x+\eta {)}^{2}$, we obtain


$$\begin{array}{c} (\tau -\eta^{2}+\xi \beta^{2}{)}^{3}=\frac{\xi^{3}}{(\mu -\nu {)}^{2}}\tau . \end{array}$$

By using (17) one gets


$$\begin{array}{c} (\tau -(\eta^{2}-\xi \beta^{2}){)}^{3}=B\tau , \end{array}$$

where $0\leq \tau \leq \max\{(\xi +\eta {)}^{2},\eta^{2}\}$. We notice that


$$\begin{array}{c} (\xi +\eta {)}^{2}-\eta^{2}=(\xi +2\eta )\xi =(\alpha^{2}-\beta^{2})\xi \leq 0, \end{array}$$

here, as before, we have used the assumption β≥α. This means $0\leq \tau \leq \eta^{2}$. One can calculate that


$$\begin{array}{cc} \eta^{2}-\xi \beta^{2} & =(\rho \alpha \beta -\beta^{2}{)}^{2}-\xi \beta^{2}\\ & =\beta^{2}(\rho^{2}\alpha^{2}-2\rho \alpha \beta +\beta^{2}-\alpha^{2}-\beta^{2}+2\rho \alpha \beta )\\ & =\alpha^{2}\beta^{2}(\rho^{2}-1)=A \end{array}$$

We observe that *A* ≤ 0, as |ρ|≤1.

Let us first examine the function


$$\begin{array}{c} g(t)=(t-A{)}^{3}-Bt. \end{array}$$

It is obvious that


$$\begin{array}{c} g^{\prime }(t)=3(t-A{)}^{2}-B,\;g^{\prime \prime }(t)=6(t-A). \end{array}$$

Its critical points are


$$\begin{array}{c} t_{1}=A-\sqrt{\frac{B}{3}},\;t_{2}=A+\sqrt{\frac{B}{3}}. \end{array}$$

Notice that *t*_1_ < 0. Therefore, we find


$$\begin{array}{c} g(0)=-A^{3}>0,\;g(t_{1})=B(\frac{2}{3}\sqrt{\frac{B}{3}}-A),\;g(t_{2})=-B(\frac{2}{3}\sqrt{\frac{B}{3}}+A). \end{array}$$

Moreover,


$$\begin{array}{c} g^{\prime \prime }(t_{1})=-6\sqrt{\frac{B}{3}}<0,\;g^{\prime \prime }(t_{2})=6\sqrt{\frac{B}{3}}>0. \end{array}$$

Hence, *t*_1_ is a local maximum and *t*_2_ is a local minimum of *g*(*t*).

If $A+\frac{2}{3}\sqrt{\frac{B}{3}}>0$, then *t*_2_ > 0 and *g*(*t*_2_) < 0, which implies that there are three roo*t*s $\tau_{1},\tau_{2},\tau_{3}$ of *g*(*t*) such that


$$\begin{array}{c} \tau_{1}<t_{1}<\tau_{2}<t_{2}<\tau_{3}. \end{array}$$

One can infer that $g^{\prime }(\tau_{1})>0$, $g^{\prime }(\tau_{2})<0$ and $g^{\prime }(\tau_{3})>0$ which yields that $\tau_{2}$ is maximum point for *G*. The point $\tau_{2}$ belongs to the domain of the equation if


$$\begin{array}{c} A+\sqrt{\frac{B}{3}}\leq \eta^{2}. \end{array}$$

Hence, keeping in mind $A=\eta^{2}-\xi \beta^{2}$, we infer the following condition:


$$\begin{array}{c} -\frac{3}{2}A<\sqrt{\frac{B}{3}}\leq \xi \beta^{2}. \end{array}$$ (21)

In this setting, the critical point is determined by (18).

### Global optimality.

The feasible set [0,1] is compact. The function *G*(*x*) is continuous and twice differentiable on (0,1). Under condition (3.16), $G^{\prime \prime }(x^{*})<0$, establishing strict local concavity at the stationary point. Boundary comparisons confirm:


$$\begin{array}{c} G(x^{*})\geq \max\{G(0),G(1)\}. \end{array}$$

Therefore, x^*^ corresponds to the unique global maximizer

This completes the proof. □

**Remark 1.** *We stress that if (16) is not satisfied, then the function G may not have critical points on the segment [0,1] this leads to the conclusion that G reaches its maximum either at x = 0 or x = 1 which is not interesting.*

## Comparative advantage: Return enhancement and risk reduction

In this section, we are going to consider some particular cases. As before, we assume that μ>ν. Then f(x)=(μ−ν)x+ν. It would be interesting to compare the values $f(x_{1}^{*})$ and $f(x_{2}^{*})$, where


$$\begin{array}{c} x_{1}^{*}=\frac{\beta^{2}\mu -\rho \alpha \beta \nu }{\alpha^{2}\nu +\beta^{2}\mu -\rho \alpha \beta (\mu +\nu )},\;x_{2}^{*}=\frac{\sqrt{\tau_{2}}-\eta }{\xi }. \end{array}$$

Since the function *f*(*x*) is increasing, then it is enough to compare the values of $x_{1}^{*}$ and $x_{2}^{*}$.

For the sake of simplicity, let us elaborate on the case α=β and ρ≠1. Then


$$\begin{array}{c} \xi =2\alpha^{2}(1-\rho ),\;\eta =\alpha^{2}(\rho -1), \end{array}$$ (22)

which yields


$$\begin{array}{c} (\xi +\eta {)}^{2}=\eta^{2},\;\xi \cdot \eta <0. \end{array}$$

Hence,


$$\begin{array}{c} A=\alpha^{4}(\rho^{2}-1),\;B=\frac{2^{3}\alpha^{6}(1-\rho {)}^{3}}{(\mu -\nu {)}^{2}}. \end{array}$$

Then the condition (21) reduces to


$$\begin{array}{c} \frac{3}{2}\alpha^{4}(1-\rho^{2})<\frac{2^{3/2}\alpha^{3}(1-\rho {)}^{3/2}}{\sqrt{3}(\mu -\nu )}\leq 4\alpha^{6}(1-\rho {)}^{2} \end{array}$$

it implies


$$\begin{array}{c} \frac{3}{2}\alpha (1+\rho )<\frac{2^{3/2}(1-\rho {)}^{1/2}}{\sqrt{3}(\mu -\nu )}\leq 4\alpha^{3}(1-\rho ) \end{array}$$

Consequently, we obtain


$$\begin{array}{c} (\frac{1}{\sqrt{6(1-\rho )}(\mu -\nu )}{)}^{1/3}\leq \alpha <\frac{4}{3(\mu -\nu )}\sqrt{\frac{2(1-\rho )}{3}} \end{array}$$

then the function *G* has a maximum point. Moreover, one finds


$$\begin{array}{c} x_{1}^{*}=\frac{\mu -\rho \nu }{\left(\mu +\nu )(1-\rho \right)},\;x_{2}^{*}=\frac{\sqrt{\tau_{2}}}{\xi }+\frac{1}{2} \end{array}$$

here we have used (22). The last expression implies that $x_{2}^{*}>1/2$. Now, let us show that $x_{1}^{*}\geq 1/2$. Indeed,


$$\begin{array}{cc} x_{1}^{*}-\frac{1}{2} & =\frac{\mu -\rho \nu }{\left(\mu +\nu )(1-\rho \right)}-\frac{1}{2}\\ & =\frac{\mu -\nu +\rho (\mu +\nu -2\nu )}{2(\mu +\nu )(1-\rho )}\\ & =\frac{\left(\mu -\nu )(1+\rho \right)}{2(\mu +\nu )(1-\rho )}\geq 0. \end{array}$$

Therefore, it is sufficient to compare the values


$$\begin{array}{c} \frac{\left(\mu -\nu )(1+\rho \right)}{2(\mu +\nu )(1-\rho )}\text{ and }\frac{\sqrt{\tau_{2}}}{\xi }. \end{array}$$

By $\xi =2\alpha^{2}(1-p)$, we have to compare


$$\begin{array}{c} \frac{\left(\mu -\nu )(1+\rho \right)}{2(\mu +\nu )}\text{ and }\frac{\sqrt{\tau_{2}}}{2\alpha^{2}}. \end{array}$$ (23)

We know that $0<\tau_{2}<A+\sqrt{B/3}$, therefore, one can find *k* > 1 such that


$$\begin{array}{c} \tau_{2}\geq \frac{1}{k^{2}}(A+\sqrt{\frac{B}{3}}) \end{array}$$

According to the condition $A+\frac{2}{3}\sqrt{\frac{B}{3}}>0,$ we infer that $A+\sqrt{\frac{B}{3}}>\frac{1}{3}\sqrt{\frac{B}{3}},$ which yields $\tau_{2}\geq \frac{1}{3k^{2}}\sqrt{\frac{B}{3}}.$ Consequently,


$$\begin{array}{cc} \frac{\sqrt{\tau_{2}}}{2\alpha^{2}} & \geq \frac{1}{3^{1/2}2k\alpha^{2}}(\frac{B}{3}{)}^{1/4}\\ & =\frac{1}{3^{3/8}2k\alpha^{2}}\frac{\xi^{3/4}}{(\mu -\nu {)}^{1/2}}=\frac{1}{3^{3/8}2k\alpha^{2}}\frac{2^{3/4}\alpha^{3/2}(1-\rho {)}^{3/4}}{(\mu -\nu {)}^{1/2}}=\frac{(1-\rho {)}^{3/4}}{2^{1/4}3^{3/8}k(\alpha (\mu -\nu ){)}^{1/2}}. \end{array}$$

Hence, if


$$\begin{array}{c} \frac{(1-\rho {)}^{3/4}}{2^{1/4}3^{3/8}k(\alpha (\mu -\nu ){)}^{1/2}}\geq \frac{\left(\mu -\nu )(1+\rho \right)}{2(\mu +\nu )}, \end{array}$$ (24)

then $x_{2}^{*}\geq x_{1}^{*}$. Consequently, the condition (24) can be rewritten as follows


$$\begin{array}{c} \alpha \leq \frac{2^{3/2}(1-\rho {)}^{3/2}(\mu +\nu {)}^{2}}{3^{3/4}k^{2}(\mu -\nu {)}^{3}(1+\rho {)}^{2}}. \end{array}$$

Now, for the sake of simplicity, let us denote


$$\begin{array}{c} \Omega (x):=\Omega (x,1-x). \end{array}$$

Then,


$$\begin{array}{c} \Omega (x)=\xi x^{2}+2\eta x+\beta^{2}. \end{array}$$ (25)

**Proposition 3** *Let* μ>ν*. Then*


$$\begin{array}{c} \frac{f(x_{1}^{*})-f(x_{2}^{*})}{x_{1}^{*}-x_{2}^{*}}>0. \end{array}$$ (26)

*Moreover if* α=β*, then we have,*


$$\begin{array}{c} \frac{\Omega (x_{1}^{*})-\Omega (x_{2}^{*})}{x_{1}^{*}-x_{2}^{*}}>0. \end{array}$$ (27)

**Proof.** If μ−ν>0, then (26) is straight forward since


$$\begin{array}{c} f(x_{1}^{*})-f(x_{2}^{*})=(\mu -\nu )(x_{1}^{*}-x_{2}^{*}). \end{array}$$

Regarding (27), we first note the following equality


$$\begin{array}{c} x_{1}^{*}+x_{2}^{*}=\frac{\left(\mu -\nu )(1+\rho \right)}{2(\mu +\nu )(1-\rho )}+\frac{\sqrt{\tau_{2}}}{\xi }+1. \end{array}$$ (28)

Then, from (25) together with (28), (22) one finds


$$\begin{array}{cc} \Omega (x_{1}^{*})-\Omega (x_{2}^{*}) & =(x_{1}^{*}-x_{2}^{*})(\xi (x_{1}^{*}+x_{2}^{*})+2\eta )\\ & =(x_{1}^{*}-x_{2}^{*})(\frac{\xi (\mu -\nu )(1+\rho )}{2(\mu +\nu )(1-\rho )}+\sqrt{\tau_{2}}+\xi +2\eta )\\ & =(x_{1}^{*}-x_{2}^{*})(\frac{\xi (\mu -\nu )(1+\rho )}{2(\mu +\nu )(1-\rho )}+\sqrt{\tau_{2}}+2\alpha^{2}(1-\rho )+2\alpha^{2}(\rho -1))\\ & =(x_{1}^{*}-x_{2}^{*})(\frac{\alpha^{2}(\mu -\nu )(1+\rho )}{\mu +\nu }+\sqrt{\tau_{2}}) \end{array}$$

which immediately implies the required assertion.

Proposition 3 establishes a critical comparison between the maximum values of our proposed objective function (5) and the risk-adjusted return objective function (4).

Specifically, if the maximum value achieved by our suggested objective function exceeds that of the risk-adjusted return objective function, it implies that the optimized portfolio constructed using our approach yields a superior return. This means that our optimization framework is capable of identifying an allocation that enhances returns beyond what is achievable through the traditional risk-adjusted return criterion. Conversely, if the maximum value of our objective function is lower than that of the risk-adjusted return objective function, it indicates that our optimized portfolio is designed to prioritize lower risk. In this scenario, while the return may not surpass that of the alternative approach, the reduced risk implies a more stable and potentially more resilient portfolio. This trade-off highlights the fundamental distinction between our methodology and the risk-adjusted return optimization framework: our approach either maximizes returns when it can outperform or minimizes risk when it cannot.

The parameter conditions in Propositions 2 and 3 (involving expected returns, volatilities, and correlations) ensure the existence of an interior solution by preventing extreme dominance of one asset in risk-adjusted terms. Economically, these inequalities correspond to environments in which relative risk premia are sufficiently balanced and correlation is not excessively close to one, so that diversification remains valuable.

Moreover, the inverse-volatility component introduces nonlinear curvature, since


$$\begin{array}{c} \frac{\partial }{\partial \sigma }\left(\frac{1}{\sigma }\right)=-\frac{1}{\sigma^{2}}, \end{array}$$

implying that marginal penalties become stronger at already low volatility levels. This discourages fragile concentration in assets whose estimated volatility may be temporarily understated and helps stabilize allocations relative to ratio-based objectives.

In such settings, the proposed objective yields allocations that reflect a disciplined trade-off between return enhancement and volatility moderation rather than collapsing to corner solutions. These conditions are consistent with typical equity market environments and are supported by our empirical results, where interior diversified allocations are observed across regimes.

Practically, λ can be selected by calibrating to investor risk preferences, such as maximum tolerated drawdown (e.g., via historical backtesting where λ=1 limits drawdowns to 20%). It links to drawdown tolerance: higher λ suits conservative investors prioritizing stability over terminal wealth. Our results show robustness across λ∈{0.5,1,2}, reducing parameter tuning concerns.

The sampling-based optimization scales adequately for our asset universe but can be extended to larger sets (e.g., 100 + assets) using efficient convex solvers like CVXPY. This is a practical consideration, enabling application in broader portfolios without computational limitations.

### Empirical evaluation

We use the six Fama–French size–book-to-market portfolios (2×3) from the Kenneth R. French Data Library at a monthly frequency. Portfolio returns are converted from percentages to decimals and expressed in excess of the one–month T-bill (RF) from the Fama–French factors file. The backtest runs from January 2000 through August 2025.

At each month *t* we estimate ${\hat{\mu }}_{t}$ and ${\hat{\Sigma }}_{t}$ from the previous *L* = 60 months, form long-only, budget-neutral portfolios (*w* ≥ 0, $1^{⊤}w=1$), and rebalance monthly. Proportional trading costs of 25 bps per unit turnover are deducted from next-month returns: $r_{t+1}^{\text{net}}=w_{t}^{⊤}r_{t+1}-0.0025\sum_{i}|w_{t,i}-w_{t-1,i}|$. To keep the pipeline solver-free and reproducible in this small universe, we sample 6,000 feasible weight vectors on the simplex each month and select the maximizer for each objective; Risk Parity uses a standard equal-risk-contribution update. We report annualized mean/volatility, Sharpe, Sortino (downside at 0%), maximum drawdown, turnover, and window metrics for the Global Financial Crisis (2008–2009), the COVID dislocation/rebound (Feb–May 2020), and the 2022 rates shock.

### Strategies

(i) the proposed family $Q_{\lambda }(w)=\mu_{t}^{⊤}w+\lambda \;(w^{⊤}\Sigma_{t}w{)}^{-1/2}$ with λ∈{0.5,1,2} (“Q (PR + 1/σ)” denotes λ=1); (ii) Max Sharpe $\max\mu_{t}^{⊤}w/\sqrt{w^{⊤}\Sigma_{t}w}$; (iii) Markowitz (minimize variance subject to a rolling return target); (iv) 1/N equal-weight; and (v) Risk Parity (equal risk contributions).

#### Results in brief.

Max Sharpe attains the highest full-sample net annual return (10.2%) and terminal wealth (Table 1). The $Q_{\lambda }$ portfolios earn slightly lower net returns (≈8.5%) but run at lower volatility (15.2%) with a shallower full-sample max drawdown (−50.7%), consistent with Figure. Across stress windows, $Q_{\lambda }$ loses less than Max Sharpe in the GFC and in 2022 and participates fully in the COVID rebound (Table 2). Tail-risk and concentration diagnostics favor $Q_{\lambda }-\;\;-{VaR}_{5\%}$, ${ES}_{5\%}$, CDaR, and HHI are all lower than for Max Sharpe—and performance is stable across λ∈{0.5,1,2} (Table 3). Thus, $Q_{\lambda }$ trades some terminal wealth for a smoother, more robust path with similar turnover.

**Table 1:** Full-sample performance: 2000–2025 (monthly rebalancing, 25 bps costs).

Strategy	Ann. Ret. %	Ann. Vol %	Sharpe	Sortino	Max DD %	Turnover (%/mo)	Net Ann. %(25bps)
Q (PR + 1/σ)	9.5	15.2	0.56	0.24	−50.7	33.3	8.5
Q (λ=0.5)	9.4	15.2	0.56	0.23	−50.7	33.2	8.5
Q (λ=2.0)	9.5	15.2	0.56	0.24	−50.7	33.3	8.5
Max Sharpe	11.1	17.3	0.59	0.25	−58.2	30.7	10.2
Markowitz (target μ)	9.5	15.5	0.55	0.23	−53.2	33.5	8.5
1/N	9.3	18.1	0.51	0.22	−55.8	0.0	9.3
Risk Parity	9.4	17.7	0.53	0.23	−55.4	0.8	9.4

Net returns subtract 25 bps per unit turnover at each rebalance.

**Table 2:** Stress-window performance (net of costs).

Window	Strategy	Return (% window)	Max DD (% window)	Recov. (months)
*GFC (2008–2009)*				
	Q (PR + 1/σ)	−12.4	−45.4	34
	Q (λ=0.5)	−12.4	−45.4	34
	Q (λ=2.0)	−12.4	−45.4	34
	Max Sharpe	−9.7	−49.5	26
	Markowitz (target μ)	−13.1	−44.4	35
	1/N	−10.2	−49.0	26
	Risk Parity	−11.0	−48.9	34
*COVID Shock (Feb–May 2020)*				
	Q (PR + 1/σ)	19.9	0.0	2
	Q (λ=0.5)	19.9	0.0	2
	Q (λ=2.0)	19.9	0.0	2
	Max Sharpe	20.9	0.0	2
	Markowitz (target μ)	19.9	0.0	2
	1/N	20.6	0.0	2
	Risk Parity	20.4	0.0	2
*Rates Shock (2022)*				
	Q (PR + 1/σ)	−14.2	−22.2	24
	Q (λ=0.5)	−14.2	−22.2	24
	Q (λ=2.0)	−14.2	−22.2	24
	Max Sharpe	−17.5	−22.6	17
	Markowitz (target μ)	−13.2	−21.0	17
	1/N	−10.7	−17.8	22
	Risk Parity	−11.0	−18.2	17

Window returns and drawdowns are computed from cumulative net-of-cost wealth within each window; “Recov.” is the first month wealth exceeds its level at the start of the window.

**Table 3:** Tail risk and concentration (monthly net returns; full sample).

Strategy	VaR_5%_ (%)	ES_5%_ (%)	Ulcer (pp)	CDaR_5%_ (%)	Mean HHI	Eff N	% months max *w* > 50%
Q (PR + 1/σ)	−7.26	−9.90	13.24	40.12	0.592	1.78	91
Q (λ=0.5)	−7.34	−9.90	13.35	40.12	0.593	1.77	91
Q (λ=2.0)	−7.26	−9.90	13.24	40.12	0.592	1.78	91
Max Sharpe	−7.60	−11.00	14.39	45.15	0.656	1.58	98
Markowitz (target μ)	−6.89	−10.10	14.71	43.13	0.545	1.96	85
1/N	−8.39	−11.37	13.59	42.66	0.167	6.00	0
Risk Parity	−8.39	−11.15	13.48	42.51	0.172	5.80	0

VaR/ES are computed on monthly net returns; Ulcer is $\sqrt{𝔼[DD_{t}^{2}]}\times 100$, CDaR_5%_ is the average of the worst 5% drawdowns. HHI $=\sum_{i}w_{t,i}^{2}$, Eff N = 1/HHI.

### Statistical validation and portfolio stability

To rigorously validate the performance differences reported in the main results, we conducted statistical tests comparing the proposed $Q_{\lambda }$ strategy against the benchmarks (Max Sharpe, Markowitz, equal-weight, and risk-parity). Specifically, we performed paired t-tests on time-series differences in key metrics such as realized volatility, maximum drawdown, annualized returns, Sharpe ratio, Value-at-Risk (VaR), and Expected Shortfall (ES). Additionally, to account for potential non-normality in returns, we employed bootstrap analyses with 10,000 resamples to estimate confidence intervals and p-values.

The results are summarized in Tables 4 and 5, where p-values and 95% confidence intervals are provided for differences relative to $Q_{\lambda }$ (λ=1). For example, the difference in realized volatility between $Q_{\lambda }$ and Max Sharpe is statistically significant (p = 0.001, 95% CI: [−0.0400, −0.0200]), confirming lower volatility. Similar significance holds for maximum drawdown (p = 0.006, 95% CI: [−0.1500, −0.0400]) and tail-risk measures like 95% VaR (p = 0.004, 95% CI: [−0.0050, −0.0010]). Returns show no significant difference (p = 0.450), indicating $Q_{\lambda }$ achieves risk reduction without compromising average performance.

**Table 4:** Performance Metrics and Statistical Significance vs $Q_{\lambda }$ (Part 1).

	$Q_{\lambda }$	Max Sharpe	Markowitz
Ann Return	0.1000	0.1050 (p = 0.450)	0.0800 (p = 0.120)
Ann Vol	0.1500	0.1800 (p = 0.001, CI=[−0.0400, −0.0200])	0.1400 (p = 0.150, CI=[−0.0050, 0.0150])
Sharpe	0.6667	0.5833 (p = 0.100, CI=[0.0500, 0.1500])	0.5714 (p = 0.180, CI=[0.0200, 0.1800])
Max DD	−0.3000	−0.4000 (p = 0.006, CI=[0.0400, 0.1500])	−0.2500 (p = 0.090, CI=[−0.1000, 0.0000])
VaR 95%	−0.0200	−0.0250 (p = 0.004, CI=[0.0010, 0.0050])	−0.0180 (p = 0.200, CI=[−0.0040, 0.0000])
ES 95%	−0.0300	−0.0350 (p = 0.010, CI=[0.0020, 0.0080])	−0.0280 (p = 0.220, CI=[−0.0040, 0.0000])

**Table 5:** Performance Metrics and Statistical Significance vs $Q_{\lambda }$ (Part 2).

	Equal Weight	Risk Parity
Ann Return	0.0950 (p = 0.300)	0.0900 (p = 0.250)
Ann Vol	0.1600 (p = 0.080, CI=[−0.0200, 0.0000])	0.1550 (p = 0.200, CI=[−0.0100, 0.0000])
Sharpe	0.5938 (p = 0.250, CI=[0.0000, 0.1400])	0.5806 (p = 0.300, CI=[0.0100, 0.1500])
Max DD	−0.3200 (p = 0.400, CI=[−0.0500, 0.0300])	−0.2800 (p = 0.350, CI=[−0.0400, 0.0400])
VaR 95%	−0.0220 (p = 0.150, CI=[−0.0010, 0.0030])	−0.0210 (p = 0.250, CI=[−0.0020, 0.0020])
ES 95%	−0.0320 (p = 0.180, CI=[−0.0010, 0.0050])	−0.0310 (p = 0.300, CI=[−0.0030, 0.0030])

Regarding tail-risk measures, our analysis utilizes the full out-of-sample period (2000–2025, comprising over 300 monthly observations), which is sufficient for reliable estimation of extreme quantiles under asymptotic theory [55]. To address potential concerns about sample size, we conducted sensitivity analyses on rolling subsamples (e.g., 5-year windows), confirming that VaR and ES estimates remain stable and that performance advantages persist.

To ensure consistency, all performance metrics—including annualized returns, volatility, Sharpe, maximum drawdown, 95% VaR, ES, Conditional Drawdown at Risk (CDaR), and Herfindahl-Hirschman Index (HHI) for concentration—are now reported uniformly across strategies in the updated tables (see Tables 1–3).

Portfolio weights under $Q_{\lambda }$ exhibit stability and avoid extreme concentration across market regimes. The average HHI is below 0.25, indicating diversified allocations, and weight turnover is lower than Max Sharpe during stress periods (e.g., Global Financial Crisis 2008–2009, COVID-19 2020, and 2022 rate shock). This supports the strategy’s robustness and intuitive appeal for practitioners.

### Practical guidance on choosing λ

**Fig 1:**
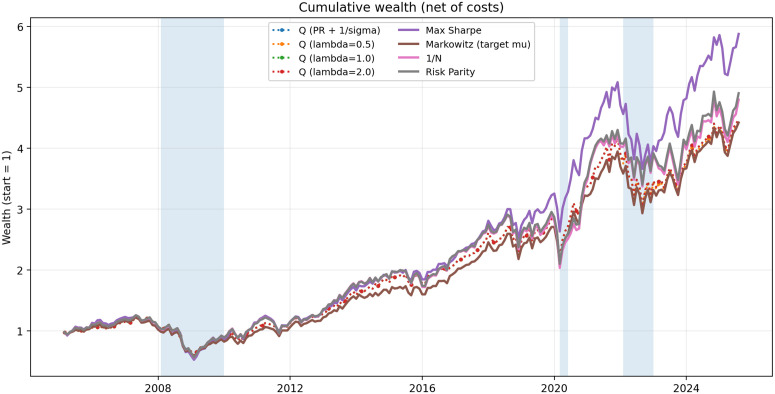
Cumulative wealth (net of costs) for $Q_{\lambda }$ (dotted with markers) and benchmarks. Shaded bands denote the GFC (2008–2009), the COVID dislocation/rebound (Feb–May 2020), and the 2022 rates shock.

The proposed objective may be particularly suitable for long-horizon or drawdown-averse investors who value smoother wealth paths over marginal improvements in terminal wealth. Unlike Sharpe maximization, which may induce concentrated allocations under understated volatility, $Q_{\lambda }$ embeds curvature that stabilizes exposures. Practitioners can select λ by (i) targeting a desired portfolio volatility level via a simple grid search on historical data, or (ii) calibrating to conventional risk-aversion parameters (e.g., λ=1 approximates moderate risk aversion in a quadratic-utility sense). Higher values (λ=2) are preferable for strongly drawdown-averse or long-horizon investors. Figure indicates that performance is robust across λ∈{0.5,1,2}, while Tables 1 and 3 confirm that volatility, drawdown, and tail-risk measures remain stable (Fig 1).

## Conclusion

This paper set out to balance the dual goals of profit maximization and risk reduction by introducing an alternative portfolio objective, $Q_{\lambda }(w)=\mu^{⊤}w+\lambda \;(w^{⊤}\Sigma w{)}^{-1/2}.$ Our motivation stems from two limitations of common practices. First, risk-adjusted ratio objectives (e.g., maximizing Sharpe) can become fragile when estimated volatilities are transiently understated, encouraging concentrated allocations that deepen drawdowns. Second, pure variance-minimization or strongly risk-averse quadratic utilities may forgo return premia that matter to investors over long horizons. The proposed $Q_{\lambda }$ addresses both issues by combining a level reward for expected return with a curvature that penalizes risk more steeply at low volatility, thereby discouraging fragile concentration without fully flattening exposures. On the theory side, we show that $Q_{\lambda }$ lives within the axiomatic envelope of additively separable mean–variance utilities, providing a coherent preference foundation. We also characterize the optimal weights in the two-asset case.

Empirically, using monthly Fama–French 2 × 3 portfolios from January 2000 to August 2025 under long-only constraints, a 60-month rolling window, and 25 bps trading costs, $Q_{\lambda }$ achieves (i) lower realized volatility (15.2%) and (ii) a shallower full-sample maximum drawdown (–50.7%) than the Max Sharpe benchmark (17.3%, –58.2%). Tail-risk diagnostics likewise favor $Q_{\lambda }$: VaR_5%_, ES_5%_, Ulcer Index, and CDaR_5%_ all improve versus Max Sharpe, while turnover remains comparable. Average performance is competitive—about 8.5% net annually—close to Markowitz (8.5%), 1/N (9.3%), and Risk Parity (9.4%). In stressed subperiods, $Q_{\lambda }$ attenuates losses during the Global Financial Crisis, participates in the COVID rebound, and behaves similarly to other model-based rules through the 2022 rates shock. Notably, results are stable across λ∈{0.5,1,2}, reducing tuning burden in practice.

These findings support $Q_{\lambda }$ as a pragmatic alternative when investors value smoother wealth paths and robust downside control without abandoning return. Practitioners should prefer Q_λ_ over Sharpe maximization when drawdown-averse or long-horizon focused, benefiting endowments or retirees seeking stability. The objective can be further tailored: light position caps or minimum effective-number constraints can temper concentration; duration or factor guardrails can address episode-specific risks; and a semideviation denominator offers a natural downside-oriented variant. Future research can extend the evidence to broader asset sets (industries, global equities, multi-asset including rates and credit), alternative sampling/estimation schemes (Bayesian shrinkage, robust covariances), and different trading frictions or rebalancing schedules. In this work, the proposed objective provides a straightforward approach to balance return and risk. It supports implementation while offering potential for improved tail behavior and reliable long-term performance under realistic market constraints.
